# Recombinant Insulin-Like Growth Factor 1 Dimers: Receptor Binding Affinities and Activation Abilities

**DOI:** 10.1007/s10989-023-10499-1

**Published:** 2023-03-04

**Authors:** Jingjing Lin, Seiya Asai, Irena Selicharová, Katarína Mitrová, Jakub Kaminský, Elinor Young, Jiří Jiráček

**Affiliations:** 1 Institute of Organic Chemistry and Biochemistry, Czech Academy of Sciences, Flemingovo nám. 2, 116 10 Prague 6, Czech Republic; 2 Department of Biochemistry, Faculty of Science, Charles University, 12840 Prague 2, Czech Republic; 3 Present Address: Department of Biology, University of York, Wentworth Way, York, YO10 5DD UK

**Keywords:** IGF-1, Receptor, Dimer, Insulin, Binding, Phosphorylation, Hormone

## Abstract

Insulin-like growth factor 1 (IGF-1) and its IGF-1 receptor (IGF-1R) belong to an important biological system that is involved in the regulation of normal growth, but that has also been recognized as playing a role in cancer. IGF-1R antagonists could be interesting for the testing of their potential antiproliferative properties as an alternative to IGF-1R tyrosine-kinase inhibitors or anti-IGF-1R monoclonal antibodies. In this study, we were inspired by the successful development of insulin dimers capable of antagonizing insulin effects on the insulin receptor (IR) by simultaneous binding to two separated binding sites and by blocking structural rearrangement of the IR. We designed and produced in *Escherichia coli* three different IGF-1 dimers in which IGF-1 monomers are interlinked through their N- and C-termini, with linkers having 8, 15 or 25 amino acids. We found that the recombinant products were susceptible to the formation of misfolded or reduced variants, but that some of them were able to bind IGF-1R in low nanomolar affinities and all of them activate IGF-1R proportionally to their binding affinities. Overall, our work can be considered as a pilot study that, although it did not lead to the discovery of new IGF-1R antagonists, explored the possibility of recombinant production of IGF-1 dimers and led to the preparation of active compounds. This work could inspire further studies dealing, for example, with the preparation of IGF-1 conjugates with specific proteins for the study of the hormone and its receptor or for therapeutic applications.

**Graphical Abstract:**

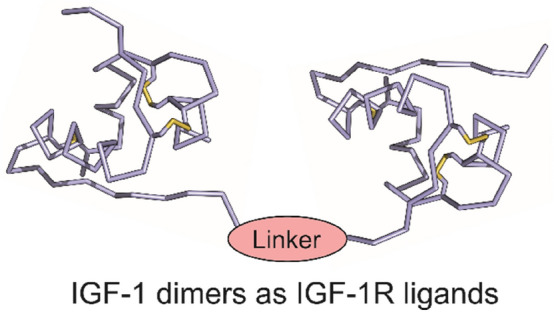

**Supplementary Information:**

The online version contains supplementary material available at 10.1007/s10989-023-10499-1.

## Introduction

Insulin-like growth factor 1 (IGF-1), together with insulin and IGF-2, belongs to a family of three related polypeptide hormones that play important roles in the human organism (Denley et al. 2005). IGF-1 (Clemmons 2009) and IGF-2 (LeRoith et al. 2021) are involved mainly in the regulation of growth processes, both during development and in adults. Insulin is responsible mainly for the uptake of glucose from blood to cells and the regulation of overall metabolic homeostasis (Belfiore et al. 2017; Saltiel 2021).

IGF-1 acts through binding to its transmembrane receptor, IGF-1R, which belongs, like both isoforms of the insulin receptor (IR-A and IR-B), to a large family of tyrosine-kinase receptors (Lemmon and Schlessinger 2010). IGF-1R, as well as IR, can be described as (αβ)_2_ dimer of two αβ monomers. Each (αβ)_2_ dimer consists of α and β chains linked by several disulfide bridges. In the *apo* form of IGF-1R (Xu et al. 2018) or IR (Croll et al. 2016; McKern et al. 2006), the two αβ monomers are thought to adopt a mutual crossover Λ-like conformation that is probably highly flexible (Gutmann et al. 2018). The α chains are only extracellular, while chains β are extracellular, transmembrane spanning and intracellular. Intracellular parts of β chains have tyrosine kinase activity and are spatially separated when the hormone is not bound to the receptor. Binding of the hormone induces a structural rearrangement in the receptor, get both kinases closer together and induce their mutual autophosphorylation. Phosphorylated tyrosine kinase subunits can transfer phosphates on intracellular proteins and trigger specific signaling cascades and biological effects of the hormone (Nagao et al. 2021).

IGF-1 is a central therapeutic target for enhancing growth retardation, muscle function in ageing and disease. Several strategies have been employed to boost its level in muscle, including transgenic expression, gene transfer or direct delivery (Barton 2006; Barton et al. 2002; Forbes et al. 2020; Lynch et al. 2001). On the other hand, IGF-1/IGF-1R signaling has been recognized as playing a role in the development of cancer (LeRoith and Roberts 2003). Several IGF-1R tyrosine-kinase inhibitors or monoclonal antibodies directed towards IGF-1R were evaluated in clinical trials (Samani et al. 2007). However, the results of these trials were rather disappointing and have not resulted in the approval of new anti-cancer drugs (Baserga 2013; Fettig and Yee 2020).

Another possible strategy for blocking the signaling of a hormone receptor is to develop compounds that bind the hormone binding site on the receptor, but do not trigger receptor activation and do inhibit the action of the native hormone. Such compounds are called antagonists if they completely inhibit receptor activity, or partial antagonists (partial agonists) if they only lower the maximum level of receptor activation. IGF-1R antagonists could be interesting for testing of their potential antiproliferative properties as an alternative to IGF-1R tyrosine-kinase inhibitors or monoclonal antibodies (Fettig and Yee 2020).

The processes of how IGF-1 or insulin bind and activate their cognate receptors are still the subject of an intensive debate and are not fully elucidated. The dimeric character of IGF-1R and IR leads to the existence of at least two equivalent binding sites for the hormone in each of the receptors.

**Fig. 1:**
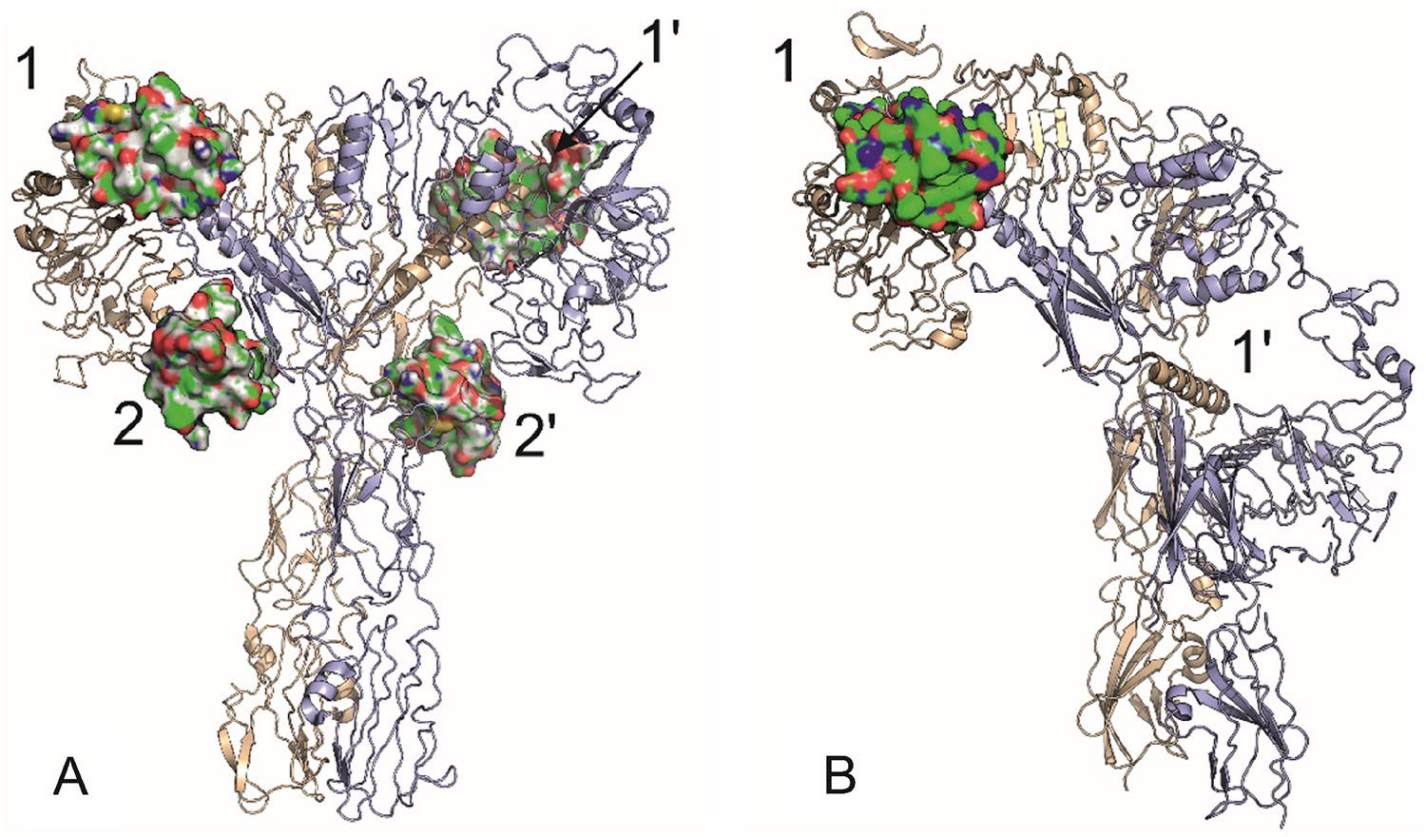
**A** Insulin receptor extracellular ectodomain (in ribbon representation with one IR protomer in magenta and the second in coral) with two insulin molecules (in surface representation) bound in Sites 1 and 1’, and two insulins bound in Sites 2 and 2’. Drawn based on the 6SOF.pdb cryo-EM structure (Gutmann et al. 2020). **B** Extracellular domain of IGF-1 receptor with one IGF-1 molecule bound to Site 1. Potential alternative Site 1’ is also labeled. Drawn based on the 6PYH.pdb cryo-EM structure (Li et al. 2019). The color scheme is the same as in **A**

The insulin:IR interaction is relatively more thoroughly studied than the related IGF-1:IGF-1R system and has provided a number of key insights. For IR, recent Cryo-EM studies showed that up to four insulin molecules can bind simultaneously to IR, which in this case adopts what is called T-shape conformation: two insulin molecules are bound in Sites 1 and 1’ and two insulins are in Sites 2 and 2’ (Gutmann et al. 2020; Li et al. 2022; Nielsen et al. 2022; Uchikawa et al. 2019) (Fig. 1A). According to this convention, Sites 1 and 2 are located on alternate αβ monomers and the same applies to Sites 1’ and 2’. It was proposed that, in the *apo* form of IR, Site 1 and Site 2 are located close together and two insulins bound in Site 1 and Site 2 would be partly overlapping (Uchikawa et al. 2019). It was also suggested that Site 2 could be the site of the first contact of the hormone with the receptor (Nielsen et al. 2022). Binding of the hormone to lower-affinity Site 2 would induce rearrangement of the receptor domains and translocation of insulin to the higher-affinity Site 1 (or binding of another insulin molecule to Site 1). This event would be followed by a separation of Site 2 from Site 1, getting receptor transmembrane b-subunits closer and activation of intracellular tyrosine kinases (Lawrence 2021; Nielsen et al. 2022). It was previously proposed that the binding of just one molecule of insulin should be sufficient for full receptor activation (Kiselyov et al. 2009) which would be consistent with low physiological concentrations of insulin (50–200 pM). Non-saturated insulin-IR complexes with 1–3 insulins bound to IR adopting various crane-like Ƭ structures were also observed (Li et al. 2022; Nielsen et al. 2022; Weis et al. 2018). It is still debated whether these unsaturated insulin:IR complexes are true activated forms of IR or whether they represent intermediate forms of IR between the *apo* Λ-form and the fully active T-form.

An interesting feature of IR is a phenomenon usually referred as a “negative cooperativity” (Levitzki and Koshland 1969). Kinetic experiments of De Meyts et al. (De Meyts et al. 1973) showed accelerated dissociation of labeled insulin by adding increasing amounts of added cold insulin (detected already for 10^−10^ M cold insulin, within the physiological range of insulin). But intriguingly, this accelerated dissociation is reduced at very high concentrations of cold insulin (above 10^−7^ M) and results in a “bell-shaped” dose-response curve (De Meyts 1994). Based on recently published Cryo-EM complexes (Gutmann et al. 2020; Li et al. 2022; Nielsen et al. 2022; Uchikawa et al. 2019; Xiong et al. 2022), these data can be interpreted as a negative cooperativity between a pair of Sites 1 and 2 and a symmetrically positioned pair of Sites 1’ and 2’ in IR; binding to Sites 1/2 weakens binding to Sites 1’/2’ (or vice versa). However, at high insulin concentrations, insulin can be simultaneously bound to both Sites 1 and 1’ or even to both Sites 2 and 2’ as shown in Fig. 1A.

Christoffersen et al. (1994) discovered that binding of IGF-1 to IGF-1R is, similarly to insulin:IR binding, characterized by a curvilinear Scatchard plot assuming two binding sites for the hormone and a ligand-accelerated tracer dissociation that is typical for a negative cooperativity. However, the negative-cooperativity curve was not “bell-shaped” as for insulin:IR interaction (De Meyts 1994), which can indicate that the simultaneous binding of two (or four) IGF-1 molecules to Sites 1 and 1’ (or 1/2 and 1’/2’) may not be possible or certainly not for a longer period. It is still not clear if IGF-1 binding to IGF-1R proceeds in a similar way as the binding of insulin to IR. Until now, Site 2 in IGF-1R has not been clearly identified and IGF-1 mutagenesis results suggest that the hormone either lacks Site 2 or that it is different from insulin’s Site 2 (Machackova et al. 2019).

Mostly only complexes with just one molecule of IGF-1 bound at Site 1 of the IGF-1R adopting asymmetric Γ shape conformation were resolved (Li et al. 2019) (Fig. 1B), which is in line with kinetic studies by Christoffersen et al. (Christoffersen et al. 1994) and receptor’s negative cooperativity property. However, two molecules of the hormone were found bound to the IGF-1R in crystals soaked with IGF-1, but this was considered as an artifact caused by constrains of the crystal lattice (Xu et al. 2018). Very recently, Moreau et al. (Moreau et al. 2022) solved cryo-EM structure (7U23.pdb) of IGF-1R construct in a pseudo-two-fold-symmetric arrangement of the receptor domains with two molecules of IGF-like viral peptide symmetrically bound to L1 domain/a-CT peptide segments (part of Site 1). Remarkably, the viral peptide behaves as natural IGF-1R antagonist and its antagonistic properties are associated with its unique C-domain sequence and Ser8 residue in the B-domain (Zhang et al. 2021), which do not allow the viral peptide to engage membrane distant part of FnIII-1’ domain (another part of Site 1). This interaction is necessary for structural transition and activation of IGF-1R. The absence of conformational transition enables binding of the second viral peptide to the alternate binding element. Interestingly, viral peptide makes sparse contacts with His539 of membrane proximal part of FnIII-1’ domain of IGF1R which is equivalent to Arg554 in IR that forms receptor’s Site 2. This would suggest that some transient Site 2 in IGF-1R, which is involved in early IGF-1 binding events, may indeed exist.

Partly antagonistic properties were also reported only for the IGF-1 analog with double Arg36-Arg37 → Glu36-Glu37 mutation (Fujita et al. 2013; Saegusa et al. 2009) at the hormone’s C-domain. Using phage display libraries, Schaeffer et al. (Pillutla et al. 2011) discovered artificial peptides that were able to antagonize either IR or IGF-1R. Brandt et al. (Brandt et al. 2018) linked some of these artificial antagonistic peptides to the position B29 in insulin, and the constructs displayed partly antagonistic properties towards IR.

Several interesting studies that were published a few decades ago by teams from Aachen in Germany (Schuttler and Brandenburg 1982; Tatnell et al. 1983; Weiland et al. 1990) inspired us in the design of the IGF-1 derivatives described in this study. They reported insulin dimers (two insulins crosslinked through B29 positions with a relatively short suberic acid) that were high-affinity partial agonists of IR. This interesting result evokes questions about the molecular mechanism by which insulin dimers can block activation of IR. Putatively, two insulins interconnected with a linker having an “optimal” length could simultaneously bind the receptor’s Sites 1 and 2 (or 1’ and 2’), block structural rearrangement of the receptor and prevent mutual contacts of the intracellular tyrosine kinase domains that are necessary for their activation. This hypothesis was recently confirmed by scientists at Merck & Co. (Pissarnitski et al. 2022), who have characterized in detail the biology of insulin dimers with partial agonistic effects towards IR. They also solved the cryo-EM structure of the IR in complex with an B29–B29-linked insulin dimer with partially antagonistic properties and were able to show that the insulin dimer units bind simultaneously to Site 1 and Site 2 in the IR and prevent complete structural rearrangement and activation of the receptor due to their connection by a short covalent linker (Wu et al. 2022). Recently we prepared B1-B1-linked insulin dimers that were IR agonists but stimulated IR more strongly than would be consistent with their binding affinities (Lin et al. 2022). These results suggest that designing peptide hormone dimers may be a promising strategy for modulating the ability of hormones to activate receptor.

The goal of this study was to probe whether IGF-1 dimers in which two IGF-1 molecules are interconnected by linkers of specific lengths can effectively bind the extracellular domain of IGF-1R and stimulate or inhibit its activation. Biological activities of such dimeric constructs could respond to some emerging questions about the mechanism of activation of IGF-1R. IGF-1 dimers having antagonistic properties towards IGF-1R could be interesting for further studies of their impact on the proliferative effects of the receptor in cellular or tissue models. Here, using a recombinant expression in *E. coli*, we prepared three different IGF-1 dimers interconnected between their C- and N-termini by linkers of different lengths. All isolated constructs were tested for binding to IGF-1R and activation of the receptor, and the results are discussed.

## Materials and methods

### Plasmid Construction

**Fig. 2:**
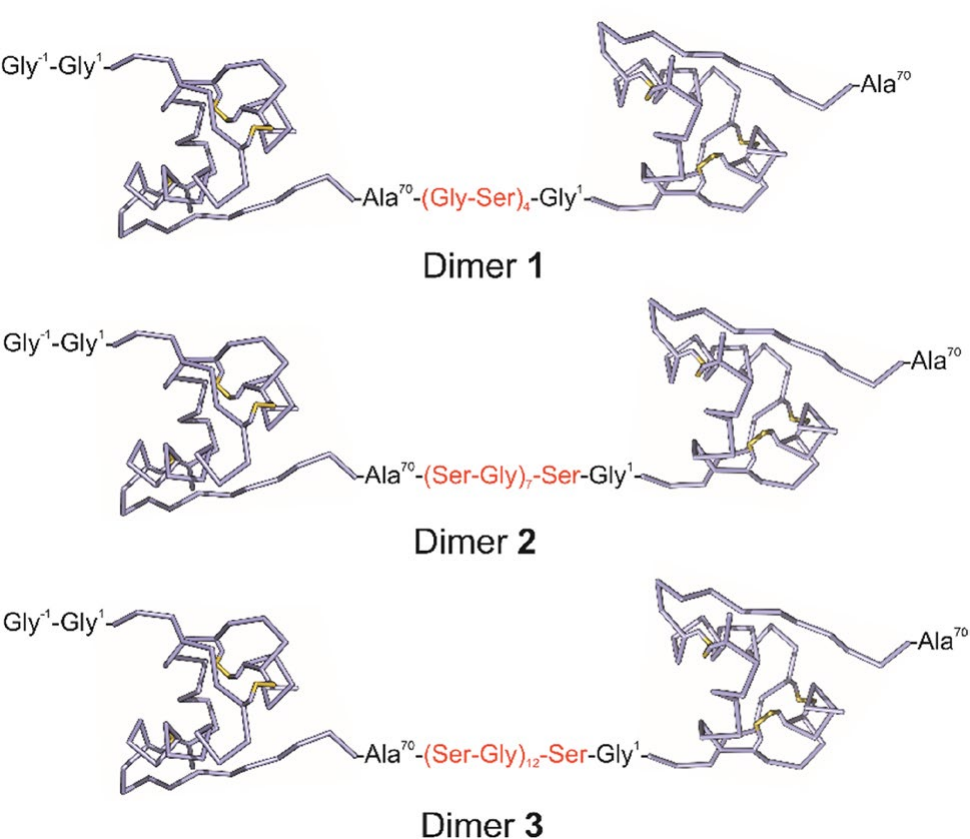
Schematic representation of IGF-1 Dimers **1**–**3** synthesized and tested in this study. IGF-1 monomers are interconnected between C-terminal Ala^70^ of the N-terminal monomer and N-terminal Gly^1^ of the C-terminal monomer with Ser-Gly chains of different length. All three constructs were prepared with an additional Gly^−1^ residue (at the position − 1) on the N-terminus. IGF-1 peptide chains are shown as blue lines, with disulfide bridges in yellow and terminal residues in black. Amino acids of the connecting linkers are shown in red

Briefly, human IGF-1 (UniprotKB entry P05019 amino acids 49–118) was cloned into a modified pRSFDuet-1 expression vector as a fusion with an N-terminally His6-tagged GB1 protein and TEV protease cleavage site. An additional N-terminal glycine residue (Gly-1) was incorporated into IGF-1 to enable cleavage (↓) by TEV protease (sequence Glu-Asn-Leu-Tyr-Phe-Gln↓Gly-1). The (Gly-Ser)_4_, (Ser-Gly)_7_-Ser or (Ser-Gly)_12_-Ser sequence in linkers connecting IGF-1 monomers (8, 15 or 25 amino acids in Dimers **1**–**3**, respectively, see in Fig. 2) were introduced at the downstream of the first IGF-1 monomer by ligating the PCR product of a vector with synthesized and annealed inserts. Vector sequence was amplified, using PCR with the primer dir (“EcoRI NotI site”) and primer rev (“IGF-1 rev wo stop codon”) (supplementary Table S1) in the presence of the template DNA (pRSFDuet-1 IGF-1 plasmid, supplementary Fig. S1) for deleting the stop-codon and for linearization. The linker inserts were prepared by annealing oligonucleotides “linker seq dir (GS)_4_”, “linker seq dir (SG)_7_S”, “linker seq dir (SG)_12_S”, “linker seq rev (GS)_4_”, “linker seq rev (SG)_7_S” and “linker seq rev (SG)_1__2_S” (supplementary Table S1). Subsequently, the vector and insert sequences were ligated and picked up to be inserted in a forward manner. Next, the latter IGF-1 sequence was connected to the downstream of the linker sequence. The latter IGF-1 sequence was PCR amplified with the primer dir (“IGF-1 dir BamHI”), and rev (“IGF-1 rev stop-codon EcoRI”) in the presence of the DNA template (pRSFD IGF-1 plasmid). Subsequently, both the vector and the insert were treated by restriction-enzymes BamHI and EcoRI, and then ligated. Identities of all constructs were verified by sequencing.

### Protein Production

The bacterial expression and purification were performed according to our previously published protocol (Hexnerova et al. 2016; Machackova et al. 2019). Constructs were transformed into *Escherichia coli* BL21 (λDE3) and grown in 1 L of LB medium, supplemented with 0.4% of glycerol and 30 µg/mL of kanamycin at 37 °C. After reaching an optical density (600 nm) of 1, the bacterial cultures were induced with 1 mM IPTG (isopropyl β-d-1-thiogalactopyranoside) and further cultured for 4–5 h. Cells were harvested by centrifugation for 20 min at 6000×*g* at 4 °C, and cell pellets (typically about 6 g) were stored at − 80 °C prior to further processing. Cell pellets containing IGF-1 peptides were resuspended in lysis buffer (50 mM Tris-HCl, pH 8.7, 50 mM NaCl, 5 mM EDTA, 0.05 mM PMSF) using 10 mL of buffer/1 g of biomass and homogenized by piston homogenizer. The suspended cells were further lysed by three passes through an Avestin EmulsiFlex-C3 apparatus at 4 °C. Inclusion bodies from the cell lysate were obtained by centrifugation at 20,000×*g* at 4 °C for 20 min and further washed as a suspension in a wash buffer (50 mM Tris-HCl, pH 8.7, 50 mM NaCl, 5 mM EDTA) in the presence 0. % (v/v) Triton X-100, sonicated in an ice bath, and centrifuged (20,000×*g*, 4 °C, 20 min). The last wash procedure was repeated, using the same buffer but without 0. % (v/v) TritonX-100, and wet paste (about 1 g) consisting of inclusion bodies was stored at − 20 °C.

Inclusion bodies were solubilized, using buffer 50 mM Tris-HCl buffer (pH 8.7) containing 300 mM NaCl, 32.5 mM β-mercaptoethanol and 8 M urea. The suspension was sonicated on ice and incubated for 2 h at room temperature with slow stirring. After being stirred for 2 h, the solubilized protein was centrifuged at 8000×*g*, 25 °C, 20 min, and the clear supernatant containing the denatured fusion protein was then loaded onto an equilibrated gravity-flow nickel chelating chromatography column (HIS-Select Nickel Affinity Gel, Sigma-Aldrich). After being washed with a buffer (50 mM Tris-HCl, pH 8.7, 300 mM NaCl), the retained protein was eluted from the column with 125 mM imidazole in 50 mM Tris-HCl (pH 8.7) buffer, supplemented with 300 mM NaCl. The presence of the fusion protein in collected fractions was analyzed by SDS-PAGE and the pooled fractions were dialyzed against 50 mM Tris-HCl (pH 8.7) buffer, supplemented with 300 mM NaCl at 4 °C for 24 h with 2 buffer exchanges. The fusion partner was subsequently cleaved by an overnight TEV digestion at room temperature in the presence of reduced (1.5 mM) and oxidized (0.15 mM) glutathione. Cleaved IGF-1 was separated (in a flow-through) from the fusion protein by gravity-flow nickel chelating chromatography (HIS-Select Nickel Affinity Gel, Sigma-Aldrich). The representative electrophoretic gel from the purification of Dimer **2** is shown in supplementary Fig. S2. The product was further desalted on a Chromabond C4 column (Macherey-Nagel) using 80% CH_3_CN (v/v) with 0.1% TFA (v/v) for elution. The collected protein fraction was lyophilized, then resuspended in 7% (v/v) acetic acid, 27% (v/v) CH_3_CN, 0.03% TFA (v/v); and purified on a semipreparative RP-HPLC column (Vydac 214TP510-C4, 250 × 10 mm) using a a gradient of CH_3_CN in H2O with 0.1% TFA (v/v). The purified IGF-1 analogs were lyophilized, and the identity of the products verified by MALDI mass spectrometry. Analytical RP-HPLC chromatograms of Dimer **2c** and Dimer **2d** fractions were measured on an analytical column (YMC-Triart-Bio C4, 250 × 4.6), using a gradient of CH_3_CN in H_2_O with 0.1% TFA (v/v).

### Binding Affinities for the Receptor

Binding affinities of analogs were determined with receptors in the intact cells. Specifically, binding affinities for IGF-1R were determined with mouse fibroblasts transfected with human IGF-1R and with deleted mouse IGF-1R, according to Hexnerová et al. (Hexnerova et al. 2016). In an ideal case (e.g. for human IGF-1 and Dimers **2c** and **2d**), the binding curve of an analog was determined in duplicate points, and the final dissociation constant (*K*_d_) was calculated from three (n = 3) binding curves (each curve giving a single *K*_d_ value), determined independently and compared with binding curves for IGF-1. However, a limited amount of isolated material in the case of Dimers **1** and **3** allowed only one measurement of a *K*_d_ value (n = 1) or even testing of the ability to displace the radiotracer at a single concentration.

### The Abilities of Analogs to Induce Autophosphorylation of the Receptor

The abilities of analogs to induce autophosphorylation of IGF-1R in membranes of mouse fibroblast transfected with human IGF-1R and with deleted mouse IGF-1R were determined, as described by Macháčková et al. (Machackova et al. 2019). Briefly, the cells were stimulated in 24-well plates (Schoeller) (4 × 104 cells per well) after 4 h of starving in serum-free medium. The cells were stimulated with 10 nM concentration of the ligands for 10 min. Stimulation was stopped by snap-freezing. Proteins were routinely analyzed, using immunoblotting and horseradish peroxidase-labeled secondary antibodies (Sigma-Aldrich). The membranes were probed with antiphospho-IGF-1Rβ (Tyr-1135/1136)/IRβ (Tyr-1150/1151) (Cell Signaling Technology). The blots were developed, using the SuperSignal West Femto maximum sensitivity substrate (Pierce), and analyzed using the ChemiDoc MP imaging system (Bio-Rad). The autophosphorylation signal density generated by each ligand on Western blotting was expressed as the contribution of phosphorylation relatively to the IGF-1 (IGF-1R fibroblasts) in the same experiment. Means were calculated from two to four independent experiments (n = 2–4), depending on the available amount of material, and were compared with native IGF-1.

### Circular Dichroism (CD) Analyses

The CD spectra of Dimers **2c**, **2d**, and native IGF-1 were recorded in solution, using the JASCO J-818 spectrometer. Proteins were dissolved in 0.1% acetic acid to the final concentration of 0.1 mg/mL. Three scans within the range of 185–270 nm were recorded and averaged to obtain the final spectra. All experiments were performed using a 1 mm quartz cell (Hellma Analytics) at room temperature, using the scanning speed of 5 nm/min, and the response time of 16 s. The signal of the solvent was subtracted from the final spectra. The absorption and CD spectra were normalized to the concentration, cell length and the number of amino acids (70 for IGF-1 and 155 for Dimers **2c** and **2d**). The secondary structure content, based on the analysis of CD spectra, was estimated using the BeStSel software (Micsonai et al. 2018). Five general secondary structures (helix, antiparallel β-sheet, parallel β-sheet, turn, and others) were considered as defined in Ref. (Micsonai et al. 2018).

### Molecular Dynamics

Two units of IGF-1 (2GF1.pdb) were in silico linked via the -(Ser-Gly)_7_-Ser- linker (extended conformation; see supplementary Fig. S3) to form Dimer **2**. Obtained structure was then placed to an orthorhombic periodic box (10 Å buffer-size) containing ~ 18,000 water molecules. The neutral charge of the box was ensured by the addition of Cl^−^ ions. After a short equilibration (100 ps, NpT ensemble), a 100 ns molecular dynamics simulation (MD; NpT ensemble) in Desmond (Schrödinger Release 2023) was performed on the box to observe the structural evolution of the dimer over time. Coordinates were recorded every 10 ps throughout the production cycle. The total energy of every tenth sampled Dimer **2** geometry (1000 in total) was calculated as implemented in Prime (Jacobson et al. 2004) (${{E}_{Total}=E}_{covalent}+{E}_{Coulomb}+{E}_{vdW}+{E}_{Solvation}$) at the OPLS4 level and the VSGB solvation model (water).

More information about molecular dynamics experiments with Dimer **2** is provided in supplementary information file.

## Results and Discussion

### Design of Compounds

IGF-1 is a single-chain protein with 70 amino acids and a relatively complicated pattern of three disulfide bridges (supplementary Fig. S4). IGF-1 has four primary amino groups: on glycine at the position 1 and on lysines at the positions 27, 65 and 68. These amines can be modified similarly to the approach of Schuttler et al. (Schuttler and Brandenburg 1982), with bi-functional linkers bearing e.g. two *N*-hydroxy-succinimide moieties. However, such reactions are not quantitative, site-selective and low yields of products can be expected. Hence, having IGF-1 in larger quantities (dozens of milligrams) would be necessary for direct chemical modifications. However, commercially available IGF-1 is rather expensive, and the chemical synthesis of IGF-1 is very difficult, with only a few attempts at the total chemical synthesis of full-length IGF-1 having been carried out (Machackova et al. 2017; Sohma et al. 2008).

Therefore, we considered a recombinant production of IGF-1 dimers. The biological production of IGF-1 allows only C- to N-terminus linkage between two IGF-1 molecules. This means that the linker must be placed between Ala70 (C-terminus) and Gly1 (N-terminus) residues. Such a linkage would correspond to the AsnB21-to-PheB1 connection in the insulin dimer, and we have not found any reference to such an insulin dimer in the literature.

We planned a recombinant production of constructs, consisting of two IGF-1 monomers linked between their C-terminus and N-terminus, with three different linkers consisting of 8, 15 or 25 amino acids. We chose Gly-Ser repeats for their flexibility and low lipophilicity. The schematic structures of the planned IGF-1 Dimers **1**–**3** are shown in Fig. 2. The linkers in Dimers **1**–**3**, in their extended conformations, should be able to bridge maximum distances of about 25, 45 and 75 Å respectively. Such linkers could be compatible e.g. with the distance (about 38 Å) that is between B1 residue of the hormone in Site 1 and A21 residue of the hormone in Site 2 of the active insulin:IR complex shown in Fig. 1A. The length of a linker could have an impact on the potential of IGF-1 dimers to modulate IGF-1R activity. It should be also noted that, in the *apo* form of IR or IGF-1R, Site 1 and Site 2 will be very probably closer than as shown in Fig. 1A that represents activated and re-arranged form of IR.

### Construction of expression plasmids for IGF-1 Dimers 1–3

IGF-1 dimers were produced in a bacterial expression system that employs the *E. coli* BL21 (λDE3) strain and the pRSFDuet-1 vector adapted for the expression of IGF-1 monomers (Hexnerova et al. 2016; Machackova et al. 2019). In this study, we first prepared three intermediate plasmids (supplementary Fig. S1), into which we inserted three different linker sequences, consisting of 8, 15 or 25 amino acids at the site placed downstream of the first IGF-1 sequence (with extra Gly residue at the position − 1 that enables cleavage of the construct by TEV protease). Then, the second IGF-1 DNA sequence (without Gly-1) was incorporated at the BamHI and EcoRI site to prepare the final plasmids. Construction of all three plasmids for expression of IGF-1 dimers were successful and the identities of the plasmids were confirmed by DNA sequencing. The experimental details are provided in “Materials and Methods” and in supplementary information.

### Purification and Identification of IGF-1 Dimer Products and Their Biological Activities

Typical purification of the IGF-1 dimer construct was done, using 1 L of LB medium in one purification batch for the growing of the transformed bacteria. Harvested cells (about 6 g of wet pellet) were processed as described in the “Materials and Methods” section. The following isolation procedures typically provided about 1-1.3 g (wet weight) of inclusion bodies.

**Fig. 3:**
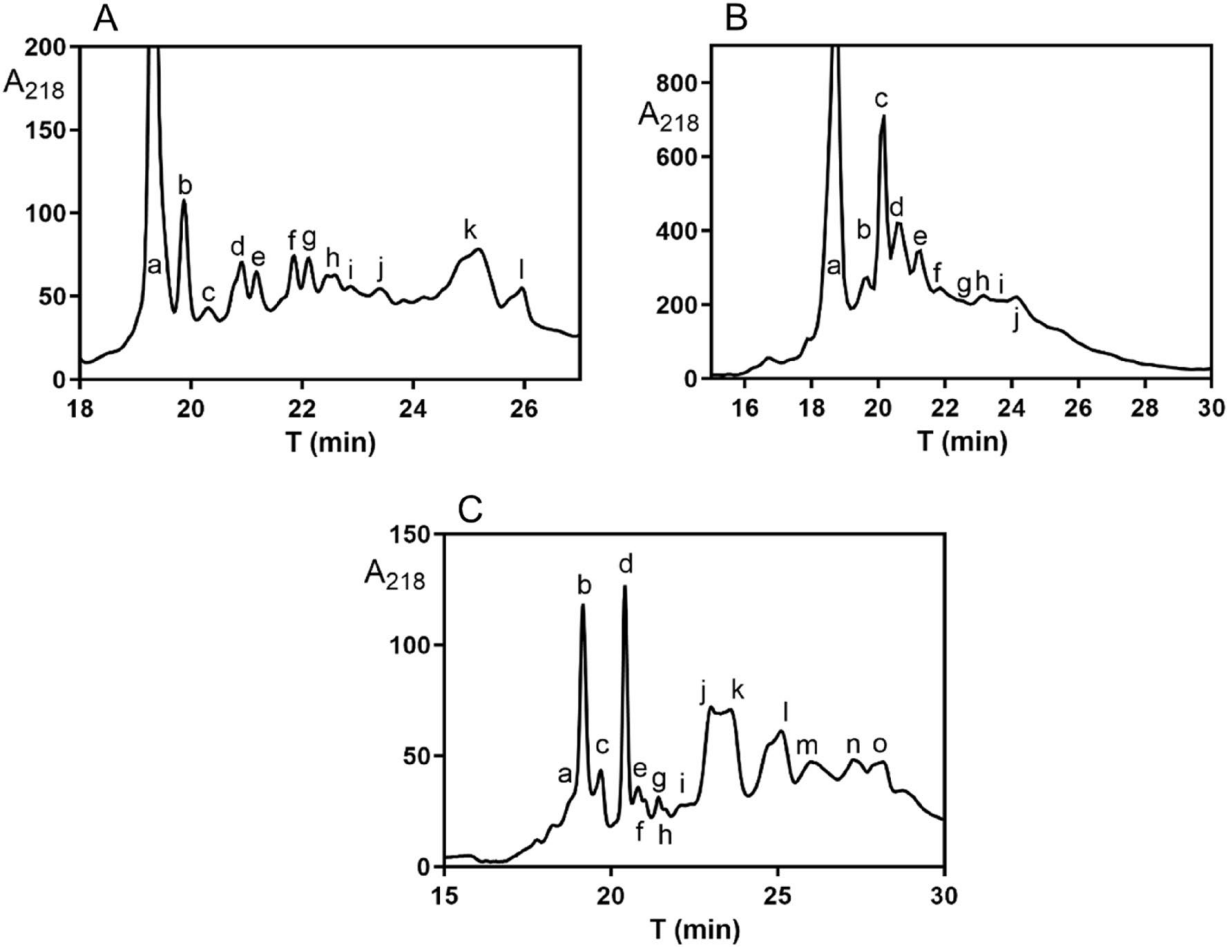
** A** RP-HPLC purification of Dimer **1** products. **B** RP-HPLC purification of Dimer **2** products. **C** RP-HPLC purification of Dimer **3** products. The m/z signals of compounds (labeled as e.g., Dimer **1a** obtained from peak a in **A** etc.) are shown in Table 1

The RP-HPLC purification of Dimer **1** products from 1.2 g of inclusion bodies resulted in isolation of 12 different fractions Dimer **1a–**Dimer **1 L** in peaks a-l (Fig. 3A). The identities of the compounds in isolated fractions were analyzed by MALDI TOF mass spectrometry and the detected m/z signals are shown in Table 1. The inaccuracy of the MALDI TOF technique for molecules of this size is at least ± 10 Da. Hence, with a reasonable probability, m/z signals 15,915 and 15,916 in peaks b, d–g and i–k could be attributed to the expected product (expected MH + mass is 15,914). Due to the low quantities of isolated material (about 10–20 µg per fraction), we were able to determine *K*_d_ values for IGF-1R for these fractions only in one measurement (n = 1). The most active fraction (**1f**) was identified in peak f and had about 21% binding affinity of thenative IGF-1. Other fractions were less active, with affinities ranging from 0.1% (for **1d**) to 4.5% (**1e**).Fractions **1j** and **k** were completely inactive. The abilities of the products to induce autophosphorylation of IGF-1R were proportional to their binding affinities (Table 2, supplementary Fig. S5).

**Table 1:** MALDI-TOF m/z signals of IGF-1 dimers detected in peaks shown in Fig. 3A-C

Fraction	m/z (MH+)	Fraction	m/z (MH+)	Fraction	m/z (MH+)
	Expected mass (average)		Expected mass (average)		Expected mass (average)
Dimer **1**	15,914	Dimer **2**	16,433	Dimer **3**	17,142

	Found		Found		Found
Dimer **1a**	No	Dimer **2a**	No	Dimer **3a**	No
Dimer **1b**	15,915	Dimer **2b**	16,433	Dimer **3b**	No
Dimer **1c**	No	Dimer **2c**	16,432	Dimer **3c**	No
Dimer **1d**	15,915	Dimer **2d**	16,432	Dimer **3d**	17,154
Dimer **1e**	15,915	Dimer **2e**	16,432	Dimer **3e**	17,156
Dimer **1f**	15,916	Dimer **2f**	16,432	Dimer **3f**	No
Dimer **1g**	15,915	Dimer **2g**	16,432	Dimer **3g**	No
Dimer **1h**	No	Dimer **2h**	16,432	Dimer **3h**	No
Dimer **1i**	15,916	Dimer **2i**	16,434	Dimer **3i**	No
Dimer **1j**	15,915	Dimer **2j**	16,435	Dimer **3j**	17,156
Dimer **1k**	15,915			Dimer **3k**	17,158
Dimer **1l**	No			Dimer **3l**	No
				Dimer **3m**	No
				Dimer **3n**	No
				Dimer **3o**	No

No means that no m/z signal that could be attributed to the expected product was found in the mass spectrum

The RP-HPLC purification of Dimer **2** products from 1 g of inclusion bodies resulted in isolation of 10 different products (**2a–2j**) from peaks a-j (Fig. 3B). The m/z mass spectrometry signals matching to the expected mass of the dimeric product were detected in all isolated fractions, except fraction **2a**. For fractions **2b** and **2f–2j**, only a few micrograms of products were obtained that allowed testing only at 10^−9^ M concentrations, where these fractions were mostly inactive.

**Table 2:** Binding affinities of human IGF-1 and IGF-1 dimers for IGF-1R and their abilities to activate this receptor

Hormone	*K*_d_ ± S.D. (nM), (n) for IGF-1R	Relative binding affinity^a^ (%)	Relative ability (at 10 nM) to induce autophosphorylation of IGF-1R (%). Mean ± S.D. (n)
Human IGF-1	0.30 ± 0.06 (5)^1^ 0.37 ± 0.03 (3)^2^ 0.26 ± 0.08 (7)^3^	100 100 100	100
Dimer **1b**	22 (1)^1^	1.4	1.2 ± 0.1 (2)
Dimer **1d**	320 (1)^1^	0.1	No
Dimer **1e**	6.6 (1)^1^	4.5	5.6 (1)
Dimer **1f**	1.4 (1) ^1^	21	23 ± 2.2 (2)
Dimer **1g**	21 (1)^1^	1.4	2.3 ± 0.3 (2)
Dimer **1i**	81 (1)^1^	0.4	No
Dimer **1j**	No binding at 10^−6^ M	–	No
Dimer **1k**	No binding at 10^−6^ M	–	No
Dimer **2b**	8% binding at 10^−9^ M	–	–
Dimer **2c**	24.2 ± 11.1 (7) (3)^3^	6.2	10 ± 1.3 (3)
Dimer **2d**	0.21 ± 0.02 (7) (3)^3^	124	100 ± 6.3 (3)
Dimer **2e**	1.3 (1)^3^	20	30 ± 3.4 (3)
Dimer **2f**	No binding at 10^−9^ M	–	No
Dimer **2g**	No binding at 10^−9^ M	–	No
Dimer **2h**	No binding at 10^−9^ M	–	No
Dimer **2i**	No binding at 10^−9^ M	–	No
Dimer **3d**	45 (1)^2^	0.8	0.2 ± 0.1 (2)
Dimer **3e**	20 (1)^2^	1.9	0.5 ± 0.2 (4)
Dimer **3j**	42 (1)^1^	0.7	–
Dimer **3k**	No binding at 10^−6^ M	–	–

Binding affinities were tested in three separate series of measurements indexed ^1^, ^2^ or ^3^

^a^Relative binding affinity is defined as (*K*_d_ of the native hormone/*K*_d_ of analog) x 100 (%)

– Means not determined. no means that no significant ability to activate the receptor was detected

The relatively higher amount of isolated material in fractions **2c** and **2d** (30–50 µg) allowed more precise determination (n = 3) of their *K*_d_ values for IGF-1R. We were able to measure at least one complete binding curve using the available material from fraction **2e**. We detected about % binding for fraction **2c** as compared with native IGF-1, about 2% binding for fraction **2e**, and fraction **2d** was equipotent to human native IGF-1. Receptor activation properties of fractions **2c**, **2d** and **2e** were generally proportional to their binding affinities (Fig. S5) without any signs of disproportional activation.

Analytical RP-HPLC chromatograms of Dimers **2c** and **2d** purified from fractions c and d are shown in the supplementary Fig. S6 and representative binding curves of these compounds for IGF-1R are shown in supplementary Fig. S7.

The RP-HPLC purification of Dimer **3** products (from 1.3 g of inclusion bodies) resulted in isolation of 15 different peaks (Fig. 3C). The m/z mass spectrometry signals that could be attributed to the expected dimer were found only in products **3d**, **3e**, **3j** and **3k** from respective peaks d, e, j and k, but these m/z signals were higher (17,154–17,156 MH^+^) than the theoretical m/z value (17,142 MH^+^). Testing for IGF-1R binding revealed binding affinities between 0.7 and 1.9% of the *K*_d_ value of the native IGF-1 for Dimers **3d**, **3e** and **3j** and no binding for Dimer **3k** at 10^−6^ M. Biological activities of Dimers **3d** and **3e** were again proportional to their binding affinities and low isolated amounts of Dimers **3j** and **3k** did not allow their testing for IGF-1R autophosphorylation. But, based on the results obtained for **3d** and **3e** we do not expect any disproportion between binding and ability to activate the receptor for **3j** and **3k** (Table 2).

Hober et al. (1992) studied different forms of IGF-1 produced in *E. coli*. They detected five major forms of the produced hormone: native, reduced or variants with mismatched pattern of disulfides. The most frequently detected misfolded variant results from a “swap” at positions Cys47 and Cys48; i.e. disulfide bridges 6–47 and 48–52 are formed instead of 6–48 and 47–52 pairs (Miller et al. 1993). The swapped variants of IGF-1 have dramatically reduced receptor binding (Machackova et al. 2017; Sohma et al. 2010). Due to the presence of 6 cysteines in the molecule, IGF-1 monomer can theoretically form up to 15 different variants of disulfide bridges. The presence of the second IGF-1 molecule can further complicate folding, and 99 different variants of disulfides are theoretically possible in one molecule of IGF-1 dimer. The number of misfolded products would be much higher if different multimers of IGF-1 connected by disulfide bridges are produced. However, we have not detected any high molecular species (like dimers of dimers) in our chromatograms. We suppose that products **3d**, **3e**, **3k** and **3j** could be partly reduced forms of IGF-1 dimers that still have several cysteines in SH forms.

Dimer **1** and Dimer **2** products had relative molecular masses detected by MALDI MS, similar to the expected fully oxidized products (Table 1), but their relative binding affinities were mostly lower than the binding affinity of native IGF-1, with the exception of Dimer **2d**. The difference between native-like binding affinity of **2d** and less active **2c** and **2e** evoked the fact that improper formation of disulfides could be behind the low binding of **2c**, **2e** and other low affinity products as well.

### Circular Dichroism (CD) Analysis of Selected Fractions

**Fig. 4:**
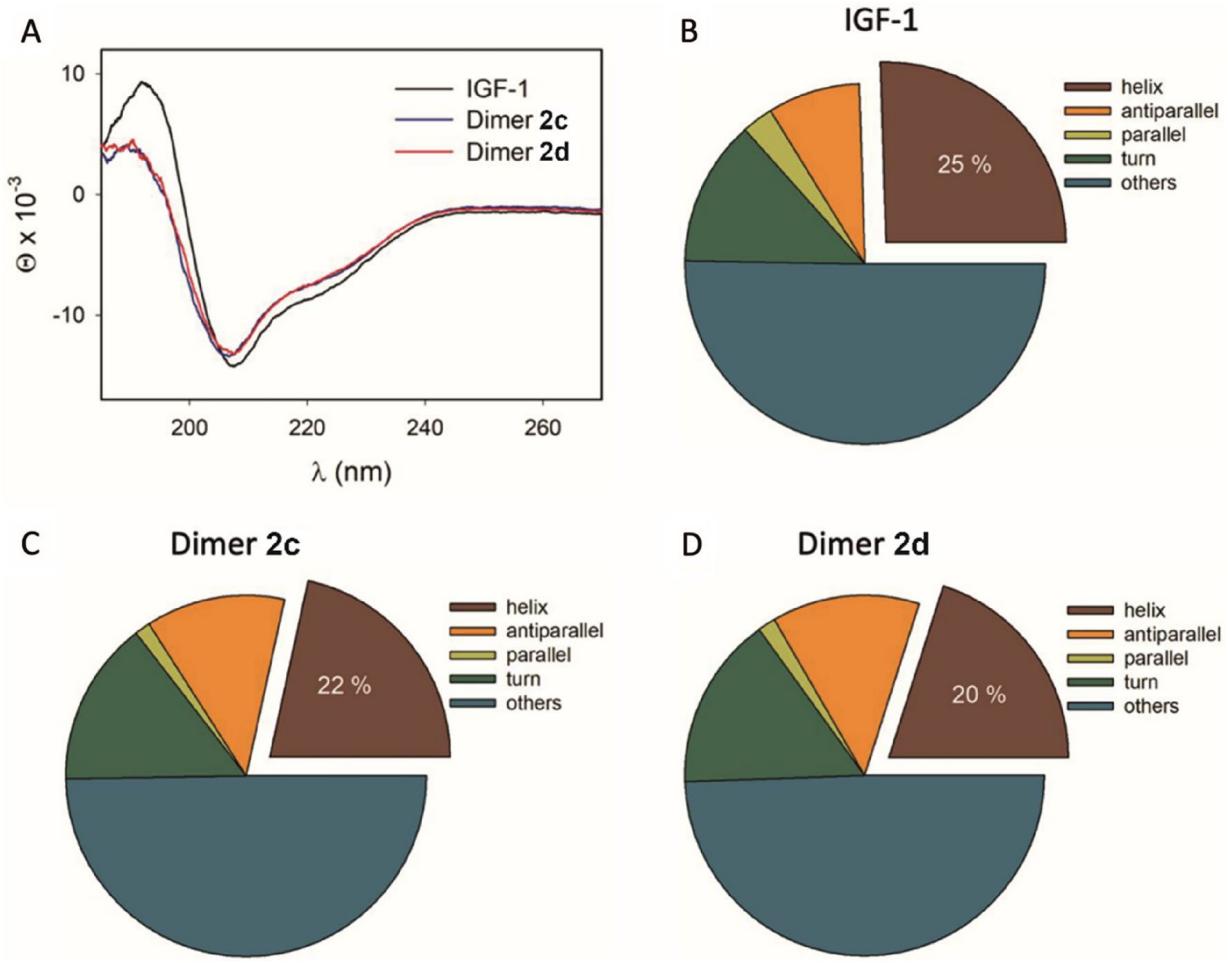
Comparison of the CD spectra of Dimers **2c** and **2d** with the native IGF-1 (**A**) and the corresponding secondary structure content for native IGF-1 (**B**), Dimer **2c** (**C**), and Dimer **2d** (D)

To investigate the possibility of the misfolding of our IGF-1 dimers, we measured the CD spectra of fractions **2c** and **2d** that have very different binding affinities for IGF-1R (6% and 124% of the native IGF-1, respectively, Table 2) and whose amount of isolated material allowed spectroscopic measurements. The results are shown in Fig. 4. Panel 4A reveals a drop of intensity of CD spectra for both dimers, compared to the spectrum of native IGF-1. The CD spectra of native IGF-1 and its misfolded (swapped) variant were previously reported by Hober et al. (1992) The swapped variant exhibited approximately three times reduced ellipticity at ~ 190 nm than native IGF-1. The observed decrease of intensity at ~ 207 and ~ 220 nm was not that significant. The authors also noticed a tiny blue shift of all bands due to misfolding. Gill et al. (Gill et al. 1999) reported the similar behavior of the CD spectra for another IGF-1/IGF-1 swap pair, yet the absolute changes differed. Our products **2c** and **2d** also exhibit lower (~ 2×) ellipticity at ~ 192 nm, as compared to native IGF-1 (Fig. 4A). The CD signals of Dimers **2c** and **2d** are practically identical. Accordingly, the estimated helical content of ~ 25% for IGF-1 drops to ~ 22% for Dimer **2c** and to ~ 20% for Dimer **2d** as seen in Fig. 4B and D. The estimated helical content ($hc$) for IGF-1 well corresponds to the content obtained from the solution NMR structure (2GF1.pdb; 27% of IGF-1. The lower estimated helical content for dimers can mostly be associated with the protein extension by the linker. When we artificially (in silico) connected two NMR structures of IGF-1 in Maestro (Maestro, Schrödinger Release 2021-3, Schrödinger, LLC, New York) by the -(Ser-Gly)_7_-Ser- linker, we could see the decrease of the helical content from ~ 27%for IGF-1 (2GF1.pdb) to ~ 25%for the construct. The helical content for the NMR structure was calculated as follows: $hc=({N}_{helix}/{N}_{all})\cdot 100$, where ${N}_{helix}$ is the number of residues corresponding to helices in the protein and ${N}_{all}$ is the number of all residues in the protein. The difference between $hc$ estimated using CD (25%) and predicted by a static mode in Maestro (22 and 20%) may be due to the error of themodel, error of the estimation by BeStSel, or due to a misfolding of dimers.

Taken altogether, the CD spectra of low active Dimer **2c** and fully active Dimer **2d** are almost identical but differ significantly from the CD spectrum of the native IGF-1. Also, the secondary structure helical content in both dimers is similar and apparently lower than in IGF-1, but this difference in helicity is likely due to the presence of the linker. On the other hand, the difference in binding affinities of both dimers that have the same linker indicates that they could differ in their tertiary structures (e.g. one or both IGF-1 units “swapped” in one of the dimers). However, we cannot detect such changes by the far-UV CD spectral analyses, and any detailed near-UV CD analysis was impossible due to the lack of a sufficient quantity of products.

## Concluding Remarks

It seems probable that all the isolated IGF-1 Dimer **1** and **2** variants, including the equipotent Dimer **2d**, can represent at least partly (in one dimer unit or in both units) misfolded variants of IGF-1. This result indicates that IGF-1 dimers linked through C and N termini can be more susceptible to the formation of misfolded products than native IGF-1, where only one misfolded variant is usually detected besides a properly folded product (Miller et al. 1993). In the case of Dimer **3** with the longest 25 amino acid linker, we detected only partly reduced forms of isolated products. We can only speculate whether the length of the linker affects the folding and formation of disulfides. It is not excluded that the longer linker in Dimer **3** promotes the formation of non-standard tertiary structures (aggregates), where at least some SH groups are not accessible to the surrounding environment and remain in a reduced form. The presence of multiple different products of each type of dimer also led to low yields of each fraction, which prevented a more thorough physicochemical characterization of the compounds.

The presence of multiple different products for each dimer type, regardless of linker type, also led to low yields of individual fractions, which prevented a more thorough physicochemical characterization of the compounds.

It worth mentioning that, despite their reduced binding affinities as compared with the native IGF-1, IGF-1 dimers prepared in this study are still able to bind IGF-1R in nanomolar concentrations and that some of the products had affinities comparable to native IGF-1 (e.g. **1f** and **2e**) or were equipotent (**2d**). This result suggests that the idea of preparing active IGF-1 dimers may be feasible, however, it will require careful optimization of production. The fact that IGF-1 is relatively tolerant, at least in some cases, to the attachment of long peptide sequences suggests that specific labeling of IGF-1 with, for example, a fluorescent tag or other chemical group would be possible. Such conjugates could be used to study the internalization of the hormone or to transport specific substances into the cell by internalization of IGF-1R.

We have not detected any marked disproportions between binding affinity to the receptor and the ability to activate the receptor for any of the tested dimeric products, and all our IGF-1 dimers, that markedly differ in the length of a connecting linker between IGF-1 units and in their binding affinities, are IGF-1R agonists. Without knowing the structure of the dimer-receptor complex, it is not easy to deduce how the dimers bind to the receptor. The distance between IGF-1 subunits in our active Dimer **2d** (cc 60-25 Å, supplementary Fig. S3) is probably not sufficient to allow efficient binding to both Sites 1 and 1’, similarly to insulins in Fig. 1A or IGF-like peptides in 7U23.pdb structure by Moreau et al. (Moreau et al. 2022), but should not preclude binding to Sites 1 and 2 as was the case for the B29-B29-linked antagonistic insulin dimers and their binding to IR (Wu et al. 2022). The lack of antagonism can be explained in different ways: the dimer does not bind to Site 1 and Site 2 simultaneously because the inherent properties of IGF-1R do not allow this in principle or the dimer binds to both sites simultaneously, but the length of its linker allows full structural transition and activation of the receptor. We think it is more likely that Dimer **2d** binds to the receptor with only one IGF-1 molecule. This could be because the N-to-C connection does not allow the same orientation of the IGF-1 molecules as the insulin molecules in the B29-B29-dimer that is necessary for an effective simultaneous targeting of Sites 1 and 2 or that one of the IGF-1 molecules is misfolded and lost its ability of potent binding.

Overall, our work can be considered as a pilot study that, although it did not lead to the discovery of new IGF-1R antagonists, explored the possibility of recombinant production of IGF-1 dimers and led to the preparation of active compounds. This work could inspire further studies dealing, for example, with the preparation of IGF-1 conjugates with specific proteins for the study of the hormone and its receptor or for therapeutic applications.

## Supplementary Information

Below is the link to the electronic supplementary material.

Supplementary material 1 (PDF 1085 kb)
