# Supplementary material for: Recombinant Insulin-Like Growth Factor 1 Dimers: Receptor Binding Affinities and Activation Abilities
Source: Int J Pept Res Ther. 2023 Mar 4;29(2):33. doi: 10.1007/s10989-023-10499-1 (PMC9985566; doi:10.1007/s10989-023-10499-1)
Supplement: Supplementary file 1 — Supplementary material 1 (PDF 1085 kb) [file 10989_2023_10499_MOESM1_ESM.pdf]

Supplementary Material

for

Recombinant insulin-like growth factor 1 dimers: receptor binding affinities and activation abilities

Jingjing Lin[1,2], Seiya Asai[1,2], Irena Selicharová[1], Katarína Mitrová[1], Jakub Kaminský[1], Elinor Young[1,3] and Jiří Jiráček[1*]

[1]Institute of Organic Chemistry and Biochemistry, Czech Academy of Sciences, Flemingovo nám. 2, 116 10 Prague 6, Czech Republic

[2]Department of Biochemistry, Faculty of Science, Charles University, 12840 Prague 2, Czech Republic

[3]Present address: Department of Biology, University of York, Wentworth Way, York YO10 5DD, United Kingdom

* Correspondence: Jiří Jiráček, jiracek@uochb.cas.cz

Supplementary Table S1. Oligonucleotide sequences prepared and used in this work for production of IGF-1 Dimers **1 - 3**. For more information, see Materials and Methods.

oligonucleotide	sequences (5’ to 3’)
linker seq dir $(GS)_4$	ggtagcggcagcggtagcggatcc
linker seq rev $(GS)_4$	ggatccgctaccgctgccgctacc
linker seq dir $(SG)_7S$	agcggcagcggtagcggcagcggtagcggtagcggcagcggatcc
linker seq rev $(SG)_7S$	ggatccgctgccgctaccgctaccgctgccgctaccgctgccgct
linker seq dir $(SG)_{12}S$	agcggcagcggtagcggtagcggcagcggtagcggcagcggtagcggcagcggtagcggtagcggcagcggatcc
linker seq rev $(SG)_{12}S$	ggatccgctgccgctaccgctaccgctgccgctaccgctgccgctaccgctgccgctaccgctaccgctgccgct
IGF-1 rev wo stop-codon	cgcgcttttcgccggtttcagc
EcoRI NotI site	taatgaattcacctgcggccgc
IGF-1 dir BamHI	catggatccggcccggaaaccctgtgcggcgcg
IGF-1 rev stop-codon EcoRI	ggtgaattcattacgcgcttttcgccgg

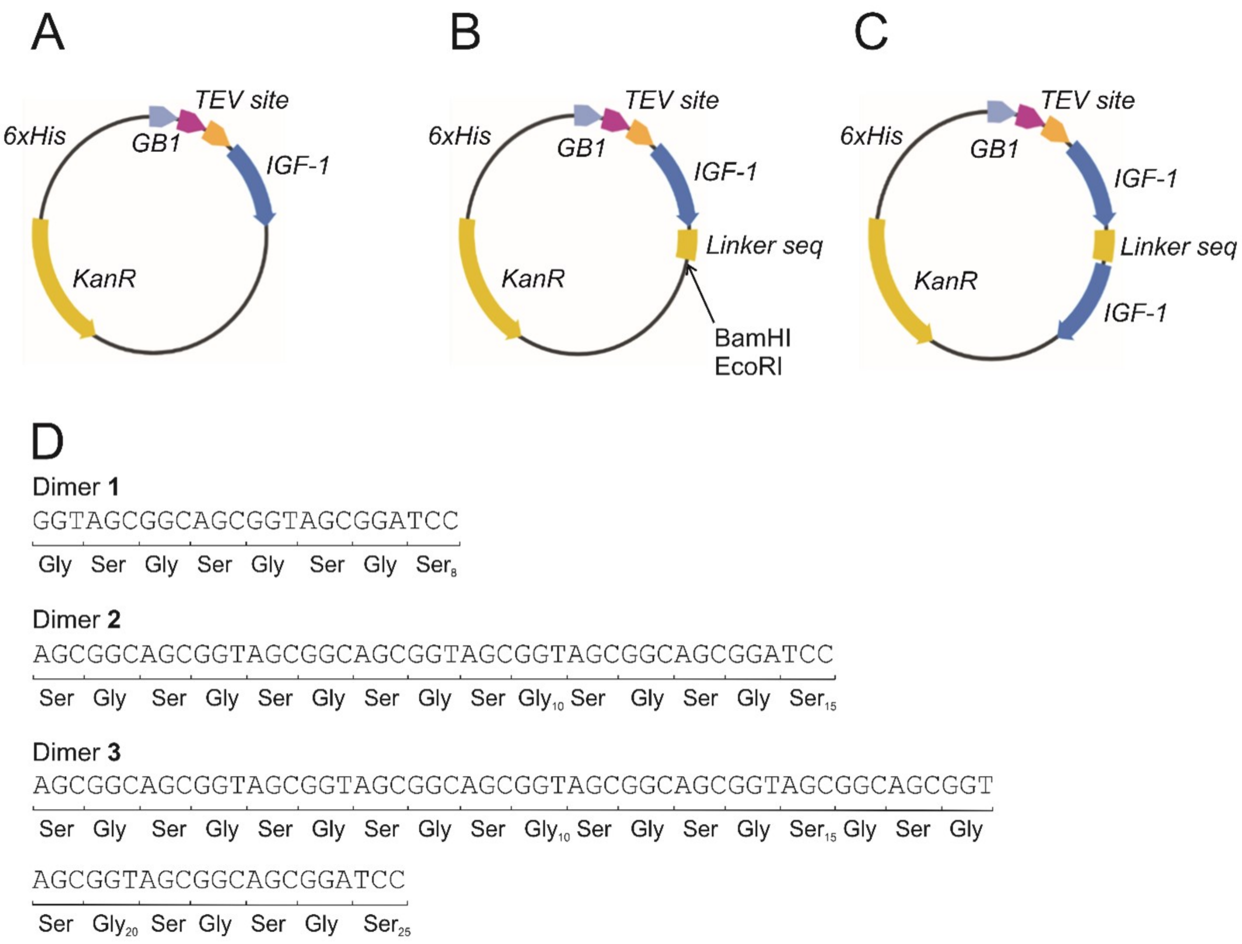

Figure S1. Schematic representation of the general strategy for the construction of the IGF-1 dimer expression plasmids. **A.** The pRSFDuet-1 plasmid adapted for the expression of IGF-1 analogs {Hexnerova, 2016 #3020;Machackova, 2019 #4728}. **B.** Three intermediate plasmids created by a ligation of the linearized original plasmid and three different linker sequences (inserted at *Linker seq* site) consisting of 8, 15 or 25 amino acids. **C.** The final plasmids into which the sequence of the second IGF-1 was inserted at the *BamHI* and *EcoRI* sites downstream of the linker sequences. *6xHis* shows the site of His-tag sequence; *GB1* is the site for immunoglobulin binding domain B1 of streptococcal Protein-G; *TEV site* is the sequence of a cleavage site for a protease from tobacco etch virus; *IGF-1* is the site for a sequence of human IGF-1 and *Linker seq* is the site for insertion of three different Ser-Gly linkers. *BamHI* and *EcoRI* are sites for recognition by respective restriction endonucleases, *KanR* is a kanamycin resistance site. **D.** Sequences of linkers in Dimers **1-3** inserted at *Linker seq* site.

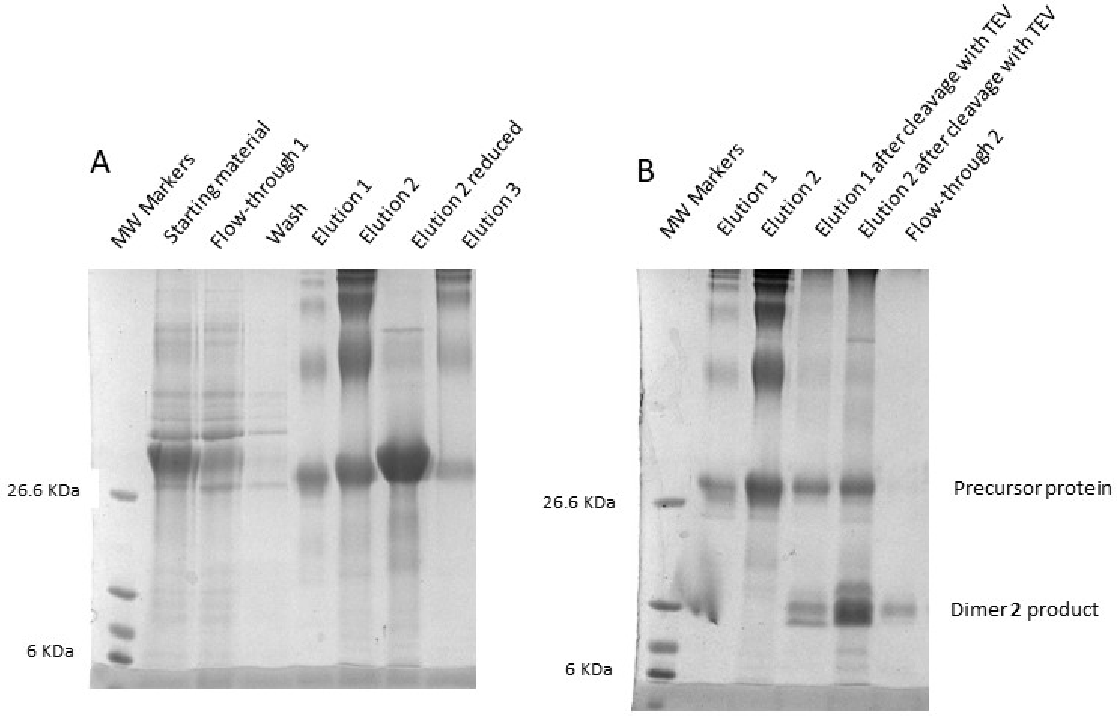

Figure S2. Polyacrylamide SDS electrophoresis of different fractions of Dimer 2 purification. **A.** *Starting material* was obtained after solubilization of inclusion bodies. *Flow-through 1* is the material that was not retained on the HIS-Select Nickel Affinity column. *Wash* is the material washed from the column with the 50 mM Tris-HCl buffer pH 8.7 with 300 mM NaCl. *Elution 1-3* is the material obtained by eluting with different (increasing) concentrations of imidazole. *Elution 2 reduced* is the same material as *Elution 2*, but after adding mercaptoethanol. **B.** *Elution 1-2* are the same fractions as in **A**, *Elution 1-2 after cleavage with TEV* are fractions treated with TEV protease and *Flow-through 2* is the material that was obtained after treating *Elution 1-2* fractions with TEV protease and that was not retained on the HIS-Select Nickel Affinity column. Approximative positions of molecular weight (MW) markers (26.6 and 6 kDa) are shown on the left. For details see Materials and Methods.

Molecular dynamics

The OPLS4 force field (1) was used to define the dimer, while water was described using the TIP3P (2). The long-range electrostatic interactions were calculated using the particle mesh Ewald method (3). The cutoff radius of Coulomb interactions was 9.0 Å. Nonbonded forces were calculated using an r-RESPA integrator. The system was heated from 0 to 300 K for 20 ps under the *NVT* conditions and equilibrated for 100 ps under the same conditions as the subsequent 100 ns production run (*NpT* ensemble at 300 K and 1 atm; Nosé–Hoover thermostat; Martyna–Tobias–Klein barostat with 2.0 ps coupling constant) (4). 10 000 geometries were equally sampled over the simulation time.

Figure S3 (see below) shows that this distance may vary from ~60 Å for extended structure to ~21 Å for more folded structure. Nevertheless, even the longer distance between the IGF-1 units in active Dimer **2** is likely shorter than the distance between Sites 1 and 1' in e.g. a crystal structure of apo IGF-1RΔβ by Xu et al. (5) By analyzing the crystal structure we can see that the shortest distance between sites is ~70 Å (effectively more). The Dimer **2** seems to prefer more folded structures with less separated IGF-1 subunits, as it is obvious from the analysis of their relative energies (**Figure S3**). Although, the predicted energies are only approximate, we see the trend that a possible transformation from the folded structure to the extended structure would be associated with relatively high energy penalty, and thus its occurrence is less probable. On the other hand, even the minimum 21 Å distance between IGF-1 units should be long enough to bridge the distance between Sites 1 and 2 (if it exists) in the apo form of IGF-1R.

1. Chao Lu, Chuanjie Wu, Delaram Ghoreishi, Wei Chen, Lingle Wang, Wolfgang Damm, Gregory A. Ross, Markus K. Dahlgren, Ellery Russell, Christopher D. Von Bargen, Robert Abel, Richard A. Friesner, Edward D. Harder. OPLS4: Improving Force Field Accuracy on Challenging Regimes of Chemical Space. J. Chem. Theory Comput. 2021, 17, 4291–4300.
2. Jorgensen, William L.; Chandrasekhar, Jayaraman; Madura, Jeffry D.; Impey, Roger W.; Klein, Michael L. Comparison of simple potential functions for simulating liquid water. J. Chem. Phys. 1983, 79, 926-35.
3. Toukmaji, Abdulnour Y.; Board, John A. Jr. Ewald summation techniques in perspective: a survey. Comput. Phys. Commun. 1996, 95, 73-92.
4. Martyna, G. J.; Klein, M. L.; Tuckerman, M. Nose-Hoover chains: The canonical ensemble via continuous dynamics. J. Chem. Phys. 1992, 97, 2635-2643.
5. Xu Yibin, Kong Geoffrey K.-W., Menting John G., Margetts Mai B., Delaine Carlie A., Jenkin Lauren M., Kiselyov Vladislav V., De Meyts Pierre, Forbes Briony E., Michael C. Lawrence. How ligand binds to the type 1 insulin-like growth factor receptor. Nat. Commun. 2018, 9: 821.

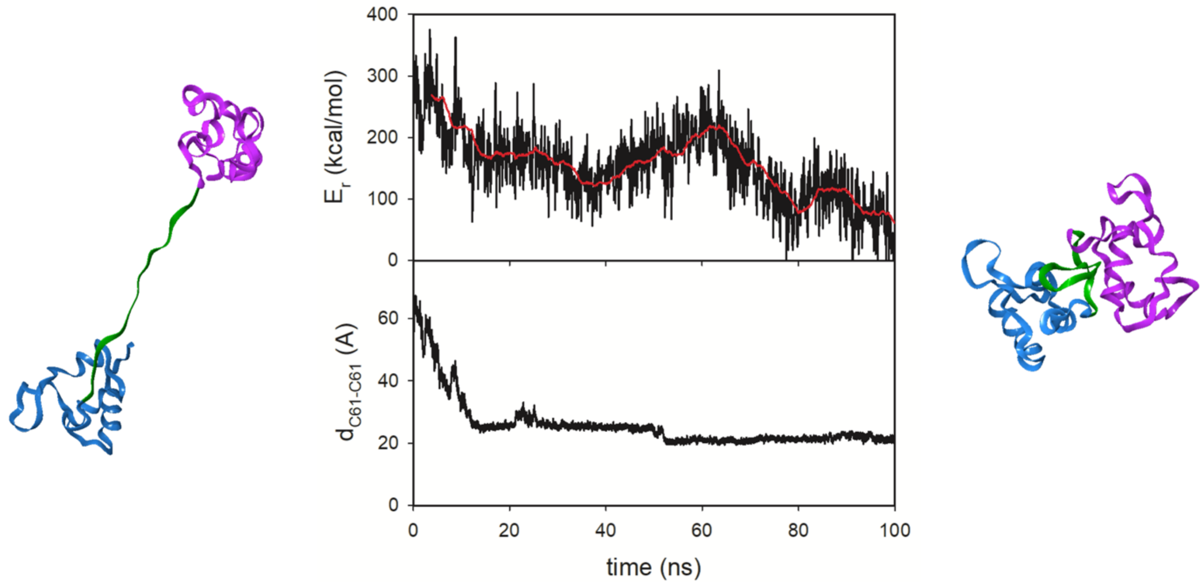

Figure S3. Time evolution of the distance between two linked IGF-1 subunits in Dimer **2** over time as provided by MD (OPLS4 force field for the protein, TIP3P for water). As a starting structure, we used a construct where two IGF-1 units were connected by the -(Ser-Gly)$_7$-Ser- linker in an extended conformation. The displayed distance represents the Cys61-Cys61 ($d_{C61\text{-}C61}$ in Å) distance, as this residue interacts (via H-bonding) with Arg705 of IGF-1R (in 6PYH.pdb). The initial 3D structure (extended) of Dimer **2** and the structure after 100 ns MD simulation are depicted on the sides. To complete the figure, the relative energies of 1000 regularly selected geometries (in kcal/mol, calculated in Prime, VSGB solvation model, OPLS4 force field) are shown. The energy of the last sampled geometry was taken as the reference. The red line shows the moving average (using the interval of 40 values).

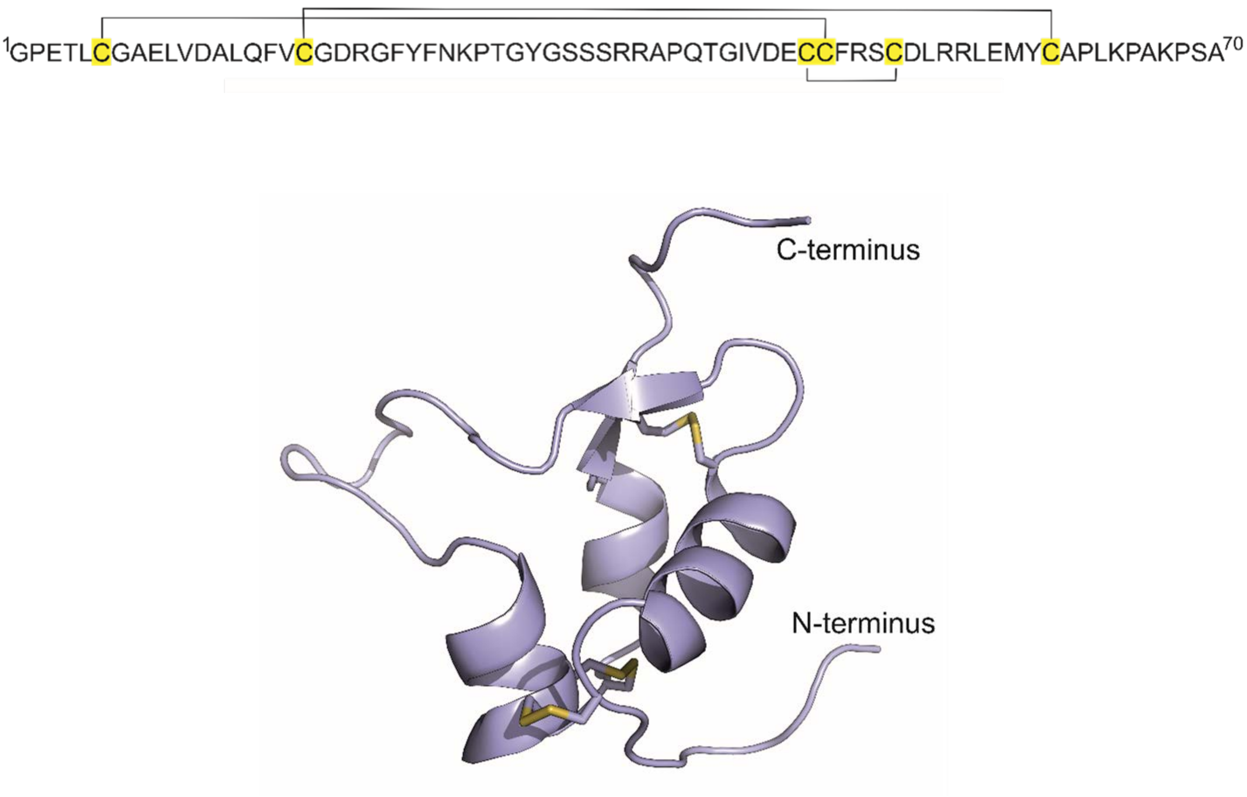

Figure S4. *Upper part:* Primary structure of human IGF-1 with schematic representation of disulfide bridges. *Lower part:* 3D-structure of IGF-1 with disulfide bridges in yellow. Prepared in PyMol according to 1IGL.pdb.

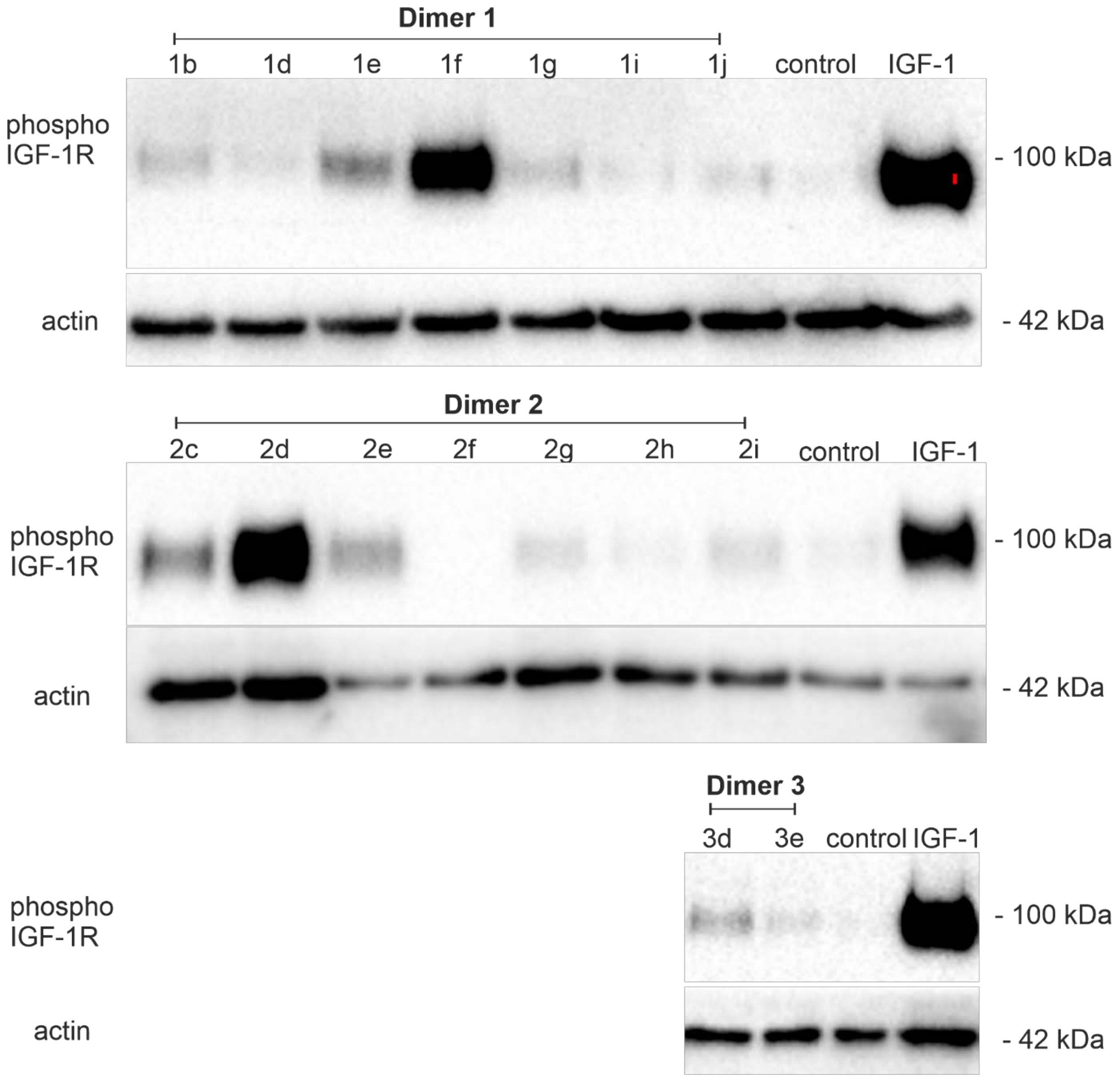

Figure S5: Representative western blots for relative abilities of IGF-1 dimers to stimulate IGF-1R phosphorylation. Cells were stimulated for 10 min with 10 nM IGF-1 dimers from individual fractions isolated using HPLC and with 10 nM IGF-1 (Tercica). Cells with no ligand were used as control. Membranes were cut at 75 kDa and 50 kDa standards and respective parts were developed with anti-phospho-IGF-1Rβ (Tyr1135/1136)/IRβ (Tyr1150/1151) antibody (Mr above 75 kDa) and with anti-actin antibody (Mr bellow 50 kDa).

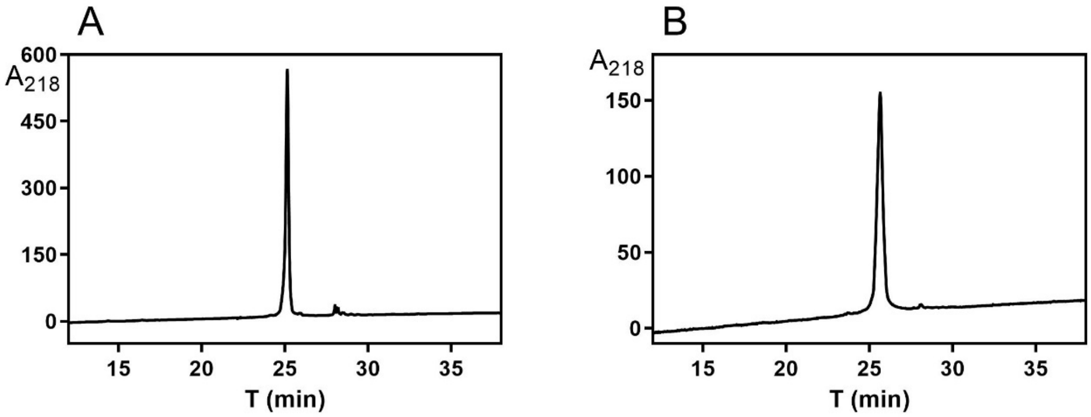

Figure S6. A. RP-HPLC analysis of isolated Dimer **2c**. **B.** RP-HPLC analysis of isolated Dimer **2d**.

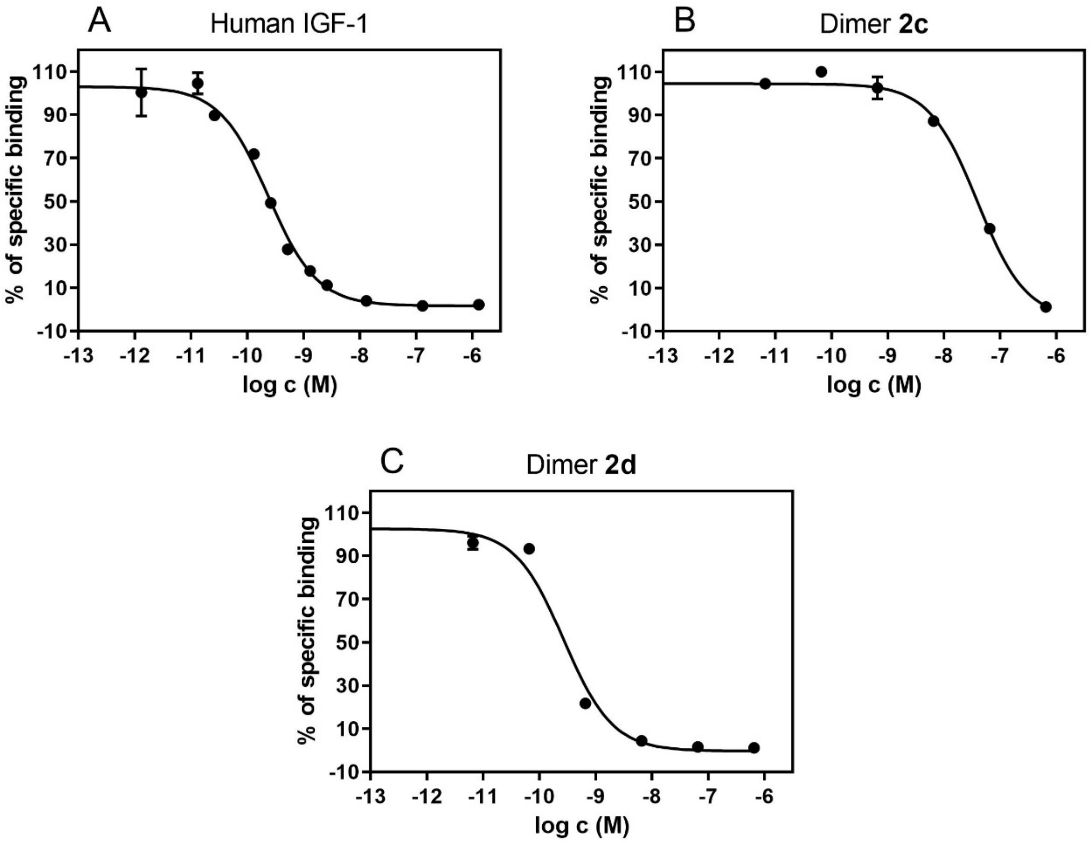

Figure S7. Representative binding curves of human IGF-1 (**A**), Dimer **2c** (**B**) and Dimer **2d** (**C**) on human IGF-1R in transfected mouse fibroblasts.
